# Association between H-type Hypertension and Asymptomatic Extracranial Artery Stenosis

**DOI:** 10.1038/s41598-018-19740-0

**Published:** 2018-01-22

**Authors:** Jia Zhang, Yanfang Liu, Anxin Wang, Dandan Wang, Ruixuan Jiang, Jiaokun Jia, Shengyun Chen, Xingquan Zhao

**Affiliations:** 1 Department of Neurology, Beijing Tiantan Hospital, Capital Medical University, Beijing, 100050 China; 2 China National Clinical Research Center for Neurological Diseases, Beijing, 100050 China; 3 Center of Stroke, Beijing Institute for Brain Disorders, Beijing, 100050 China; 4 Beijing Key Laboratory of Translational Medicine for Cerebrovascular Disease, Beijing, 100050 China; 5 Department of Epidemiology and Health Statistics, School of Public Health, Capital Medical University, Beijing, 100050 China

## Abstract

Asymptomatic extracranial artery stenosis (ECAS) is a well-known risk factor for stroke and H-type hypertension, which is defined as hypertension with hyperhomocysteinemia, is associated with cardio-cerebrovascular diseases. However, the impact of H-type hypertension on ECAS is mostly unknown. We designed this study to investigate the association between H-type hypertension and prevalence of ECAS. We included 2330 participants in this study and classified them into four groups: the control group without hypertension or hyperhomocysteinemia, isolated hypertension group, isolated hyperhomocysteinemia group and H-type hypertension group. We measured the baseline plasma total homocysteine levels and assessed ECAS by carotid duplex sonography twice at baseline and during follow up. We used a Cox regression model to analyse the association between H-type hypertension and ECAS. At baseline, 608 subjects suffered from H-type hypertension. Within two years of follow-up, asymptomatic ECAS occurred in 250 (10.73%) participants. After adjusting for relevant risk factors, we found H-type hypertension to be an independent risk factor for asymptomatic ECAS (relative risk (RR) 3.16, 95% confidence interval (95% CI) 2.00–5.00). Our findings provide direct evidence for the importance of H-type hypertension in the occurrence of ECAS and as a potential therapeutic target for carotid atherosclerosis.

## Introduction

Cardio-cerebrovascular diseases, such as atherothrombotic events, are major causes of death throughout the world every year^1^, and carotid atherosclerotic disease is an important risk factor for cardio-cerebrovascular disease^2–4^. Extracranial artery stenosis (ECAS) is one of the most common contributors to ischemic stroke, which accounts for approximately 15% of stroke cases^5^. A previous study showed that ECAS affects approximately 7% of women and more than 12% of men in the elderly^6^. This finding has a critical effect on public health and requires early detection and prevention. In China, the prevalence of hypertension is 29.6%^7^, and approximately 75% of hypertensive patients have elevated homocysteine levels^8^. Some researchers have found that hypertension and hyperhomocysteinemia are both risk factors for cardio-cerebrovascular disease and that their effects are synergistic^9,10^. Therefore, H-type hypertension, which is defined as hypertension with an elevated level of plasma homocysteine (≥10 µmol/L)^11^, has received increasing attention in recent years and has become a focus of research. However, few studies have assessed the association between H-type hypertension and the risk of ECAS. We designed this prospective study to investigate the impact of H-type hypertension on the prevalence of asymptomatic ECAS. This study is aimed at screening the risk factors for ECAS and helping to prevent cardiovascular disease at an early stage.

## Results

### Baseline characteristics

Our study included 2330 participants who were divided into four groups. There were 608 participants (26.09%) in the H-type hypertension group, 938 participants (40.26%) in the isolated hyperhomocysteinemia group, 217 participants (9.31%) in the isolated hypertension group and the remaining 567 participants (24.33%) in the control group at baseline. Participants with ECAS were more likely to be male and older age, smoke and drink, have a medical history of diabetes mellitus (DM) and dyslipidaemia, and have a higher body mass index (BMI) than control participants. We also found them to have a high occurrence of H-type hypertension (Table 1).

**Table 1:** Clinical baseline characteristics of participants according to ECAS.

	Total	ECAS		P
		None (n = 2080)	New-onset (n = 250)	
Age, year	47.63(43.87, 54.00)	47.09(43.57, 53.37)	53.23(47.16, 60.60)	<0.001
Sex, n%				
Female	1252(53.73)	1156(55.58)	96(38.40)	<0.001
Male	1078(46.27)	924(44.42)	154(61.60)	
Smoking, n%				
Never	1630(69.96)	1476(70.96)	154(61.60)	0.004
Former	66(2.83)	54(2.60)	12(4.80)	
Current	634(27.21)	550(26.44)	84(33.60)	
Drinking, n%				
Never	1653(70.94)	1505(72.36)	148(59.20)	<0.001
Former	16(0.69)	13(0.63)	3(1.20)	
Current	661(28.37)	562(27.02)	99(39.60)	
Diabetes mellitus, n%				
Yes	165(7.08)	138(6.63)	27(10.80)	0.019
No	2165(92.92)	1942(93.37)	223(89.20)	
Dyslipidaemia, n%				
Yes	987(42.36)	847(40.72)	140(56.00)	<0.001
No	1343(57.64)	1233(59.28)	110(44.00)	
BMI, n%				
Ideal	1276(54.76)	1157(55.63)	119(47.60)	0.037
Overweight	900(38.63)	785(37.74)	115(46.00)	
Obese	154(6.61)	138(6.63)	16(6.40)	
Classifications of HT and HHCY, n%				
H-type hypertension	608(26.09)	511(24.57)	97(38.80)	<0.001
Isolated HT	217(9.31)	194(9.33)	23(9.20)	
Isolated HHCY	938(40.26)	838(40.29)	100(40.00)	
Without HT and HHCY	567(24.33)	537(25.82)	30(12.00)	

*Data are presented as median (25% interquartile range, 75% interquartile range) or N(%).

ECAS: extracranial artery stenosis; HT: hypertension; HHCY: hyperhomocysteinemia; BMI: body mass index.

### Prevalence of ECAS

During the 2-year follow-up, we identified 250 participants developed ECAS. 38.80% (97/250) of subjects in the H-type hypertension group, 9.20% (23/250) in the isolated hypertension group, 40.00% (100/250) in the isolated hyperhomocysteinemia group and 12.00% (30/250) in the control group developed ECAS.

### Correlation between H-type hypertension and ECAS

In univariate analysis, baseline isolated hypertension (relative risk (RR) 2.51, 95% confidence interval (95% CI) 1.46–4.33), isolated hyperhomocysteinemia (RR 3.63, 95% CI 2.39–5.53) and H-type hypertension (RR 6.28, 95% CI 4.11–9.61) were all associated with the presence of ECAS during follow-up. In multivariate analysis, we adjusted for age, sex, smoking and drinking status, diabetes mellitus, dyslipidaemia and BMI and found that H-type hypertension was an indicator of ECAS (multivariate-adjusted RR 3.16, 95% CI 2.00–5.00). We observed a clear relationship between H-type hypertension and the occurrence of ECAS (P < 0.05, Table 2).

**Table 2:** RRs of ECAS for classifications of hypertension and hyperhomocysteinemia at baseline.

HT	HHCY	Unadjusted		Adjusted 1		Adjusted 2	
category		RR(95% CI)	P	RR(95% CI)	P	RR(95% CI)	P
No	No	1.00 (reference)	−	1.00 (reference)	−	1.00 (reference)	−
Yes	No	2.51 (1.46–4.33)	0.001	1.98 (1.14–3.43)	0.015	1.74 (0.99–3.04)	0.052
No	Yes	3.63 (2.39–5.53)	<0.001	2.34 (1.51–3.61)	<0.001	2.31 (1.49–3.58)	<0.001
Yes	Yes	6.28 (4.11–9.61)	<0.001	3.36 (2.15–5.26)	<0.001	3.16 (2.00–5.00)	<0.001

^*^RR: relative risk; 95% CI: 95% confidence interval; ECAS: extracranial artery stenosis; HT: hypertension; HHCY: hyperhomocysteinemia.

Adjusted 1: Adjusted for age, sex.

Adjusted 2: Adjusted for age, sex, status of smoking and drinking, histories of DM and dyslipidemia, BMI.

## Discussion

In our study population, the prevalence of asymptomatic ECAS was 10.73%. We observed isolated hypertension, isolated hyperhomocysteinemia and H-type hypertension were associated with the incidence of asymptomatic ECAS, especially H-type hypertension. Like other traditional risk factors, such as age, DM and dyslipidaemia, H-type hypertension is an independent risk factor for the development of asymptomatic ECAS. Our study is the first to focus on the predictive impact of H-type hypertension on asymptomatic ECAS.

There have been several studies demonstrating the relationship between H-type hypertension and atherosclerotic carotid diseases. Zhang *et al*. found that hyperhomocysteinemia combined with hypertension may increase the degree of early carotid artery atherosclerosis compared to elevated homocysteine levels with normal blood pressure. The fully adjusted odds ratios (ORs) were 1.67 (95% CI 1.15–2.42) for increased carotid intima-media thickness and 2.48 (95% CI 1.54–3.99) for bilateral plaques^12^. A large population-based retrospective study showed that the detection rate of carotid atherosclerotic plaques in patients with H-type hypertension was significantly higher than in those with isolated systolic hypertension or elevated homocysteine^13^. Using a multivariable logistic regression analysis, the authors found that a significant association remained between H-type hypertension and the incidence of carotid atherosclerotic plaques. Another study that enrolled 987 subjects reported that H-type hypertension was associated with the recurrence of ischemic stroke^14^. Our findings are consistent with those studies. In our cohort, isolated hypertension, isolated hyperhomocysteinemia and H-type hypertension had an effect on the occurrence and development of ECAS, and the association between H-type hypertension and ECAS remained statistically significant after adjusting for other relevant risk factors. We conclude that H-type hypertension is an independent risk factor for ECAS and that H-type hypertension provides predictive value for ECAS. Most of the previous studies analysed the predictive role of H-type hypertension for cerebrovascular diseases or mild carotid artery atherosclerotic diseases, such as carotid intima-media thickness or plaques. There are few studies focused on H-type hypertension and asymptomatic carotid artery atherosclerotic disease, especially asymptomatic ECAS. To the best of our knowledge, this study is the first community-based, prospective, long-term follow-up study to assess the potential relationship between H-type hypertension and the incidence of asymptomatic ECAS. Our study suggests that H-type hypertension is an independent risk factor for asymptomatic ECAS, on which more attention should be focused. Early recognition and treatment of H-type hypertension are necessary to delay the progression of carotid atherosclerosis and help prevent stroke.

The predictive role of hypertension for carotid atherosclerosis has been widely proven^15,16^, and inflammation may be the essential mechanism. Hypertension through vasoactive peptides promotes and accelerates the atherosclerotic process^17^. Although the relationship between hyperhomocysteinemia and atherosclerotic carotid diseases is controversial^12,18–21^, there have been studies demonstrating the potential mechanisms by which hyperhomocysteinemia contributes to the development of atherosclerosis, such as increased oxidative stress, impaired endothelial function, and alterations of lipid metabolism, to promote the formation of atherosclerotic plaques as well as increase the adhesion of platelets and induction of thrombosis^22^. In addition, the synergistic effects of hypertension and hyperhomocysteinemia may be explained by the fact that hyperhomocysteinemia activates the angiotensin-converting enzyme by inhibiting the production of endogenous hydrogen sulfide to lead to or aggravate hypertension^23–25^. Therefore, when hypertension and hyperhomocysteinemia are combined, the effects on atherosclerosis may be increased.

The potential limitations in our study need to be acknowledged. First, folate, vitamin B12, and pyridoxal-5′-phosphate are related to the metabolism of homocysteine, and several previous studies have found that they may be associated with an increased risk of vascular disease. However, in our study, we only tested the levels of plasma homocysteine. In future follow-up studies, we plan to add these parameters and expect to further demonstrate the relationship between the abnormal metabolism of homocysteine and atherosclerotic carotid diseases. Second, ECAS was evaluated by duplex sonography, and the testing accuracy was lower than angiography. In addition, the sonography results may be operator dependent to some extent. However, sonography is noninvasive, convenient and the most commonly used method to detect carotid diseases worldwide. Sonography is more appropriate than computed tomography angiography (CTA), magnetic resonance angiography (MRA) and digital subtraction angiography (DSA) in large cohort screening. Furthermore, an ultrasound specialist team was consulted if there was any uncertainty about the ultrasound results to reduce detection bias in our study. Third, the population under study in our cohort was Asian and middle-aged to elderly subjects. Thus, more studies are needed to confirm the generalizability of our findings to other ethnicities and races or to younger subjects.

In conclusion, we observed a clear relationship between H-type hypertension and the presence of asymptomatic ECAS. Our findings provide direct evidence of the importance of H-type hypertension in the development of ECAS and as a potential therapeutic target for carotid atherosclerosis.

## Methods

### Study design and participants

**Figure 1:**
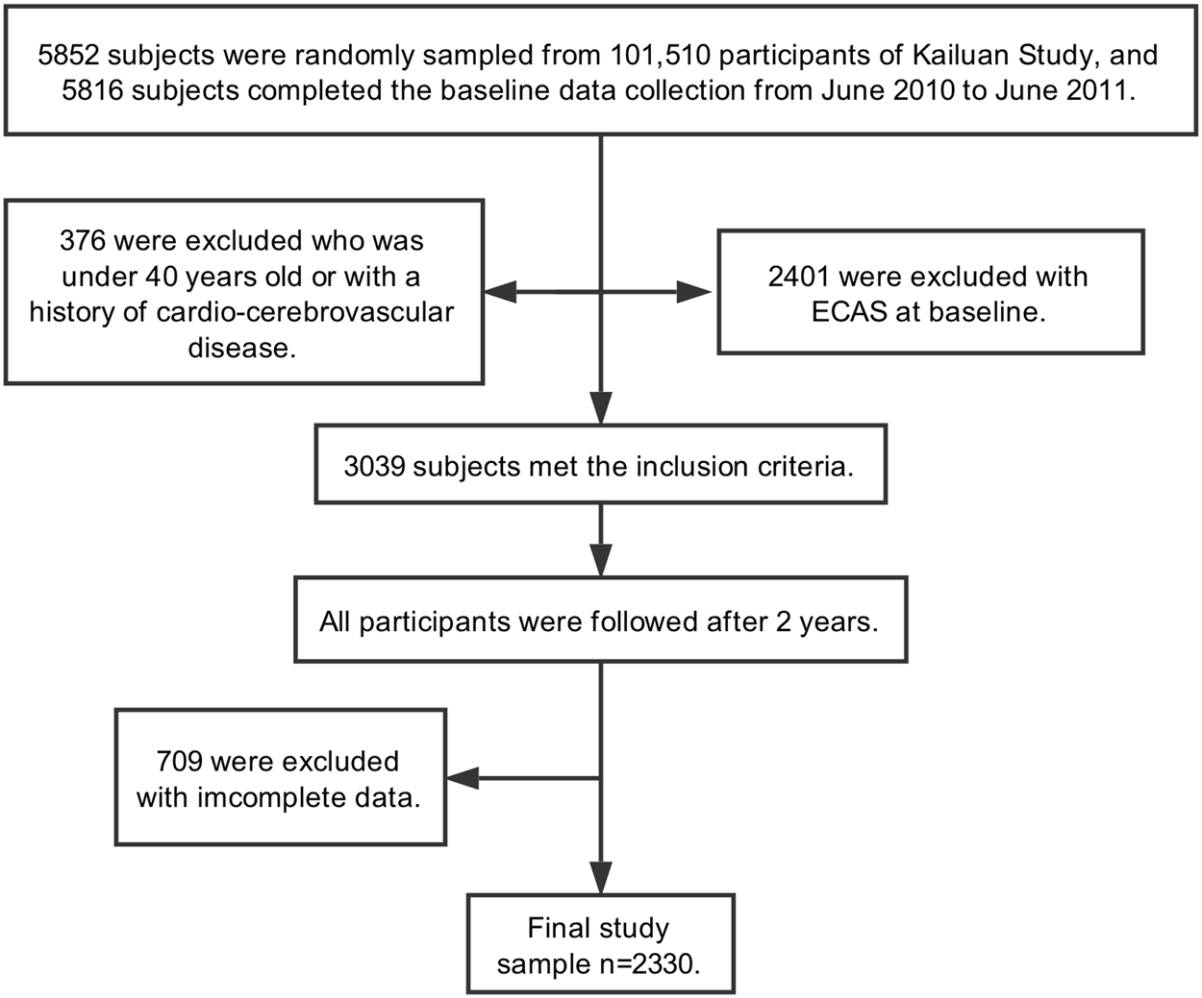
Flowchart of the stud.

The Asymptomatic Polyvascular Abnormalities Community (APAC) study is a community-based, prospective, long-term follow-up study that is designed to investigate the epidemiology of asymptomatic polyvascular abnormalities in Chinese adults. The inclusion criteria were as follows: (1) no history of stroke, transient ischemic attack or coronary heart disease at baseline as assessed by a validated questionnaire; (2) the absence of neurologic deficits for stroke, which was examined by experienced physicians. Detailed information on the study design and population has been described in our previous published protocol^26,27^. Briefly, a total of 5852 subjects older than 40 years were randomly sampled from the Kailuan cohort of 101 510 participants (81 110 males and 20 400 females, aged 18–98 years old) from June 2010 to June 2011. Of these subjects, we obtained baseline data for 5816. After excluding 376 subjects who did not meet the inclusion criteria, a total of 5440 participants were finally included in the study. All the subjects underwent a questionnaire, clinical assessment, laboratory assessment and carotid duplex sonography at baseline as well as attended a follow-up per biennium to undergo carotid duplex sonography. In our study, 2401 subjects with ECAS were excluded during the baseline investigation and 3039 subjects without ECAS were selected, of whom 709 were excluded because of incomplete data, leaving 1252 women and 1078 men (total of 2330 subjects) in this analysis (Fig. 1). The study was performed according to the guidelines from the Helsinki Declaration and was approved by the Ethics Committees of the Kailuan General Hospital and the Beijing Tiantan Hospital. Written informed consent was obtained from all participants.

### Independent variable definition and classification

Hypertension was defined as the presence any of the following: a self-reported history or any use of anti-hypertensive medications, systolic blood pressure ≥140 mmHg, or diastolic blood pressure ≥90 mmHg. Hyperhomocysteinemia was defined as the level of plasma homocysteine ≥10 µmol/L. H-type hypertension was defined as hypertension with an elevated level of plasma homocysteine (≥10 µmol/L)^11^. All of the participants were classified into four groups according to whether they suffered from hypertension or hyperhomocysteinemia: (1) the control group: without hypertension and hyperhomocysteinemia, (2) isolated hypertension group: hypertension without hyperhomocysteinemia, (3) isolated hyperhomocysteinemia group: hyperhomocysteinemia without hypertension, and (4) H-type hypertension group: both hypertension and hyperhomocysteinemia.

### ECAS assessment

Bilateral extracranial arteries were evaluated with a professional duplex sonography (Philips iU-22 ultrasound system, Philips Medical Systems, Bothell, WA). Every participant underwent carotid sonography of the bilateral common carotid arteries, internal carotid arteries, external carotid arteries, vertebral arteries and subclavian arteries at baseline and during following-up. The severity of arterial stenosis was classified into five degrees according to the diameter of the artery, namely <50% stenosis, 50–69% stenosis, ≥70% stenosis to near occlusion, near occlusion, and total occlusion. These values were based on the recommendations from the Society of Radiologists in Ultrasound Consensus Conference. If the extracranial artery diameter measurements were not available, ECAS was defined by the peak systolic blood flow velocity (PSV): (1) <50% stenosis when the ICA PSV was less than 125 cm/s and the plaque or intimal thickening was visible; (2) 50–69% stenosis when the ICA PSV was 125–130 cm/s and the plaque was visible; (3) ≥70% stenosis to near occlusion when the ICA PSV was greater than 230 cm/s and a visible plaque and lumen narrowing were seen; (4) near occlusion when there was a markedly narrowed lumen on colour Doppler US; and (5) occlusion when there was no detectable patent lumen on grey-scale US and no flow on spectral, power and colour Doppler US^28,29^. In our study, we diagnosed ECAS when one or more of the following arteries, including the extracranial common carotid artery, internal carotid artery and vertebral artery were observed to be stenotic to any degree by ultrasound.

### Potential covariates assessment

Every participant completed a standardized questionnaire to collect information on age, sex, medical history and other basic information^28^. Age was analysed as a covariate including a cut-off value of 60 years for older age. We classified BMI into ideal (BMI <25 kg/m^2^), overweight (BMI ≥  25 kg/m^2^ but <30 kg/m^2^) or obese (BMI ≥ 30 kg/m^2^). Smoking and drinking status were classified as “never”, “former”, or “current” according to self-reported information. DM was diagnosed by any of the following: fasting blood glucose level ≥7.0 mmol/L, a self-reported history of DM, or current treatment with insulin or oral hypoglycemic agents. Dyslipidaemia was defined as the presence of any of the following: a self-reported history, current treatment with lipid-lowering medications, LDL-C ≥3.37 mmol/L, HDL-C <1.04 mmol/L, triglycerides ≥1.7 mmol/L, or total cholesterol ≥5.17 mmol/L^30,31^.

### Statistical analysis

Statistical analysis was performed using the SAS software (version 9.1; SAS Institute, Cary, North Carolina, USA). As all continuous variables were non-normally distributed, they are presented as the median and inter-quartile ranges. Categorical variables are presented as the frequencies and percentages. Continuous variables were compared using Wilcoxon or Kruskal-Wallis test, and categorical variables were compared using chi-squared tests. We used Cox regression analysis to determine the associations between the presence of ECAS and H-type hypertension and calculate the relative risks (RRs) and 95% confidence intervals (CIs). All of the statistical tests were two-sided, and a P-value <0.05 was considered to be statistically significant.

### Data availability

The datasets analysed in the current study are available from the corresponding author on reasonable request.

## Electronic supplementary material

Supplementary Information

## References

[1] Lee WH (2014). Cardiovascular Events in Patients with Atherothrombotic Disease: A Population-Based Longitudinal Study in Taiwan. PloS one.

[2] Petty GW (1999). Ischemic stroke subtypes: a population-based study of incidence and risk factors. Stroke.

[3] Good, E*. et al*. High-grade carotid artery stenosis: A forgotten area in cardiovascular risk management. *European Journal of Preventive Cardiology* **23** (2016). 10.1177/2047487316632629 26879568

[4] Perk, J. *et al*. European Guidelines on cardiovascular disease prevention in clinical practice (version 2012). *The Fifth Joint Task Force of the European Society of Cardiology and Other Societies on Cardiovascular Disease Prevention in Clinical Practice (constituted by rep*. (W.B. Saunders Company, 2012). 10.1007/s12529-012-9242-5 23093473

[5] Kolominsky-Rabas PL, Weber M, Gefeller O, Neundoerfer B, Heuschmann PU (2001). Epidemiology of ischemic stroke subtypes according to TOAST criteria: incidence, recurrence, and long-term survival in ischemic stroke subtypes: a population-based study. Stroke.

[6] Raman G (2013). Management strategies for asymptomatic carotid stenosis: a systematic review and meta-analysis. Annals of internal medicine.

[7] Wang J, Zhang L, Wang F, Liu L, Wang H (2014). Prevalence, awareness, treatment, and control of hypertension in China: results from a national survey. American journal of hypertension.

[8] Qin X (2012). Effect of folic acid intervention on the change of serum folate level in hypertensive Chinese adults: do methylenetetrahydrofolate reductase and methionine synthase gene polymorphisms affect therapeutic responses?. Pharmacogenetics and genomics.

[9] Trabetti EH (2008). MTHFR gene polymorphisms, and cardio-cerebrovascular risk. Journal of applied genetics.

[10] Ganguly P, Alam SF (2015). Role of homocysteine in the development of cardiovascular disease. Nutrition Journal.

[11] Qin X, Huo Y (2016). H-Type hypertension, stroke and diabetes in China: Opportunities for primary prevention. Journal of diabetes.

[12] Zhang Z (2016). Combined Effect of Hyperhomocysteinemia and Hypertension on the Presence of Early Carotid Artery Atherosclerosis. Journal of stroke and cerebrovascular diseases: the official journal of National Stroke Association.

[13] Chen, Z., Wang, F., Zheng, Y., Zeng, Q. & Liu, H. H-type hypertension is an important risk factor of carotid atherosclerotic plaques. *Clinical and experimental hypertension* (*New York*, *N.Y*.: *1993*) **38**, 424**–**428, 10.3109/10641963.2015.1116547 (2016). 10.3109/10641963.2015.1116547 27359263

[14] Zhang, Q*. et al*. H-Type Hypertension and C Reactive Protein in Recurrence of Ischemic Stroke. *International journal of environmental research and public health* **13**, 10.3390/ijerph13050477 (2016).

[15] Ebrahim S (1999). Carotid plaque, intima media thickness, cardiovascular risk factors, and prevalent cardiovascular disease in men and women: the British Regional Heart Study. Stroke.

[16] Dua A (2012). Asymptomatic 50% to 75% internal carotid artery stenosis in 288 patients: risk factors for disease progression and ipsilateral neurological symptoms. Perspectives in vascular surgery and endovascular therapy.

[17] Li JJ, Chen JL (2005). Inflammation may be a bridge connecting hypertension and atherosclerosis. Medical hypotheses.

[18] McCully KS, Ragsdale BD (1970). Production of arteriosclerosis by homocysteinemia. The American Journal of Pathology.

[19] Selhub J (1995). Association between plasma homocysteine concentrations and extracranial carotid-artery stenosis. The New England journal of medicine.

[20] Okura T (2014). Hyperhomocysteinemia is one of the risk factors associated with cerebrovascular stiffness in hypertensive patients, especially elderly males. Scientific reports.

[21] Linnebank M (2006). Homocysteine and carotid intima-media thickness in a german population: lack of clinical relevance. Stroke.

[22] Spence JD (2007). Homocysteine-lowering therapy: a role in stroke prevention?. The Lancet Neurology.

[23] Mendis S, Athauda SB, Naser M, Takahashi K (1999). Association between hyperhomocysteinaemia and hypertension in Sri Lankans. Journal of International Medical Research.

[24] Laggner H (2007). The novel gaseous vasorelaxant hydrogen sulfide inhibits angiotensin-converting enzyme activity of endothelial cells. Journal of Hypertension.

[25] Antoniades C, Antonopoulos AS, Tousoulis D, Marinou K, Stefanadis C (2009). Homocysteine and coronary atherosclerosis: from folate fortification to the recent clinical trials. European heart journal.

[26] Zhou Y (2013). Asymptomatic polyvascular abnormalities in community (APAC) study in China: objectives, design and baseline characteristics. PloS one.

[27] Wu S (2012). Prevalence of ideal cardiovascular health and its relationship with the 4-year cardiovascular events in a northern Chinese industrial city. Circulation. Cardiovascular quality and outcomes.

[28] Wang D (2015). Arterial pre-hypertension and hypertension in intracranial versus extracranial cerebrovascular stenosis. European journal of neurology.

[29] Grant EG (2003). Carotid artery stenosis: gray-scale and Doppler US diagnosis–Society of Radiologists in Ultrasound Consensus Conference. Radiology.

[30] Wang J (2014). Associations of high sensitivity C-reactive protein levels with the prevalence of asymptomatic intracranial arterial stenosis. European journal of neurology.

[31] Wang A (2016). Carotid intima-media thickness and cognitive function in a middle-aged and older adult community: a cross-sectional study. Journal of Neurology.

