# Supplementary material for: Association between H-type Hypertension and Asymptomatic Extracranial Artery Stenosis
Source: Sci Rep. 2018 Jan 22;8:1328. doi: 10.1038/s41598-018-19740-0 (PMC5778020; doi:10.1038/s41598-018-19740-0)
Supplement: Supplementary file 1 — Supplementary Information [file 41598_2018_19740_MOESM1_ESM.pdf]

Title page

Association between H-type Hypertension and Asymptomatic Extracranial Artery Stenosis

Jia Zhang[a,b,c,d*], Yanfang Liu[a,b,c,d*], Anxin Wang[a,b,c,d,e], Dandan Wang[a,b,c,d], Ruixuan Jiang[a,b,c,d], Jiaokun Jia [a,b,c,d], Shengyun Chen[a,b,c,d]†, Xingquan Zhao[a,b,c,d]†

[a]Department of Neurology, Beijing Tiantan Hospital, Capital Medical University, Beijing, 100050, China.

[b]China National Clinical Research Center for Neurological Diseases, Beijing, 100050, China.

[c]Center of Stroke, Beijing Institute for Brain Disorders, Beijing, 100050, China.

[d]Beijing Key Laboratory of Translational Medicine for Cerebrovascular Disease, Beijing, 100050, China.

[e]Department of Epidemiology and Health Statistics, School of Public Health, Capital Medical University, Beijing, 100050, China.

All these authors take responsibility for all aspects of the reliability and freedom from bias of the data presented and their discussed interpretation.

† Corresponding authors: Prof. Xingquan Zhao, Department of Neurology, Beijing Tiantan Hospital, Capital Medical University, 6 Tiantan Xili, Dongcheng District, Beijing, China, 100050. Phone: 86-10-67098471. Fax: 86-10-67013383. Email: zxq@vip.163.com; Prof. Shengyun Chen, Department of Neurology, Beijing Tiantan Hospital, Capital Medical University, 6 Tiantan Xili, Dongcheng District, Beijing, China, 100050. Phone: 86-10-67098121. Fax: 86-10- 67096699. Email: csywindy@163.com.

*First authors: Jia Zhang (email: emjiazh@163.com) and Yanfang Liu (email: liuyanfang@126.com) contributed equally to this work.

Supplementary Table S1 Baseline characteristics of participants and non-participants

	All	Participants (n=2330)	Non-participants (n=709)	P
Age,year	48(43.96, 54.37)	47.63(43.87, 54.00)	49.63(44.40, 56.20)	<0.001
Sex, n%				
Female	1564(51.46)	1252(53.73)	312(44.01)	<0.001
Male	1475(48.54)	1078(46.27)	397(55.99)	
Smoking, n%				
Never	2102(69.17)	1630(69.96)	472(66.57)	0.155
Former	93(3.06)	66(2.83)	27(3.81)	
Current	844(27.77)	634(27.21)	210(29.62)	
Drinking, n%				
Never	2125(69.92)	1653(70.94)	472(66.57)	0.041
Former	25(0.82)	16(0.69)	9(1.27)	
Current	889(29.25)	661(28.37)	228(32.16)	
Diabetes mellitus, n%				
Yes	237(7.80)	165(7.08)	72(10.16)	0.010
No	2802(82.20)	2165(92.92)	637(89.84)	
Dyslipidaemia, n%				
Yes	1297(42.68)	987(42.36)	310(43.72)	0.544
No	1742(57.32)	1343(57.64)	399(56.28)	
BMI, %				
Ideal	1617(53.21)	1276(54.76)	341(48.10)	0.006
Overweight	1209(39.78)	900(38.63)	309(43.58)	
Obese	213(7.01)	154(6.61)	59(8.32)	
Classifications of HT, n%				
Without HT and HHCY	733(24.12)	567(24.33)	166(23.41)	
Isolated HT	301(9.90)	217(9.31)	84(11.85)	0..001
Isolated HHCY	1175(38.66)	938(40.26)	237(33.43)	
H-type HT	830(27.31)	608(26.09)	222(31.31)	

*Data are presented as median (25%interquartile range, 75% interquartile range) or N (%).

HT: hypertension; HHCY: hyperhomocysteinemia; BMI: body mass index
